# Consequences of Repeated Defoliation on Belowground Bud Banks of *Carex brevicuspis* (Cyperaceae) in the Dongting Lake Wetlands, China

**DOI:** 10.3389/fpls.2016.01119

**Published:** 2016-07-29

**Authors:** Xin-Sheng Chen, Zheng-Miao Deng, Yong-Hong Xie, Feng Li, Zhi-Yong Hou, Chao Wu

**Affiliations:** ^1^Key Laboratory of Agro-ecological Processes in Subtropical Region, Institute of Subtropical Agriculture, The Chinese Academy of SciencesChangsha, China; ^2^Dongting Lake Station for Wetland Ecosystem Research, Institute of Subtropical Agriculture, The Chinese Academy of SciencesChangsha, China

**Keywords:** bud bank, clonal growth, clonal plant, disturbance, grazing, population regeneration

## Abstract

Despite the predominant role of bud banks in the regeneration of clonal macrophyte populations, few studies have examined the way in which clonal macrophytes adjust the demographic features of bud banks to regulate population dynamics in response to defoliation in wetlands. We investigated the density and composition of bud banks under repeated defoliation in the wetland sedge *Carex brevicuspis* C. B. Clarke in the Dongting Lake wetlands, China. The density and biomass of rhizome buds and shoots did not decrease significantly in response to repeated defoliation over two consecutive years. The composition of bud banks, which consisted of long and short rhizome buds, also did not change significantly in response to repeated defoliation. Nevertheless, the ramet height and the shoot, root, and rhizome mass of *C. brevicuspis* declined significantly under repeated defoliation. Our findings suggest that bud banks are a conservative reproductive strategy that enables *C. brevicuspis* to tolerate a certain amount of defoliation. The maintenance of large bud banks after repeated defoliation may enable *C. brevicuspis* populations to regenerate and persist in disturbed habitats. However, bud bank density of *C. brevicuspis* might decline in the long term because the amount of carbon stored in rhizome buds and plants is reduced by frequent defoliation.

## Introduction

Defoliation by herbivores or mowing is a common disturbance in ecosystems dominated by perennials, such as grasslands and wetlands (Zhao et al., 2008; Terer et al., 2012). In these ecosystems, clonal plants reproduce predominantly from a belowground population of meristems, the “bud bank” (Harper, 1977; Benson et al., 2004; Benson and Hartnett, 2006; Sosnová et al., 2010; Ott and Hartnett, 2012; Deng et al., 2015). Tillering from bud banks is one of the major mechanisms conferring plant resilience to herbivory (Tuomi et al., 1994; Strauss and Agrawal, 1999; Tiffin, 2000). Therefore, the population dynamics of clonal species in response to defoliation may be determined by the demographic features of bud banks, such as the number of buds available for tiller recruitment and their emergence rate (Tuomi et al., 1994; Huhat et al., 2000; Lehtilä, 2000; Dalgleish and Hartnett, 2009).

The effect of defoliation on bud bank demography differs among plant guilds (Vesk, 2006; Dalgleish and Hartnett, 2009; VanderWeide and Hartnett, 2015). For example, defoliation by grazers increases grass bud banks but decreases forb bud banks in tallgrass prairies (Dalgleish and Hartnett, 2009). In addition, the number of buds available for tiller recruitment fluctuates over the year (Benson et al., 2004; Dalgleish and Hartnett, 2006; Zhang et al., 2009; Chen et al., 2014, 2015b,c). Therefore, the effect of defoliation on bud bank demography may also vary among seasons.

Buds in the bud bank may be classified according to their size, developmental stage, location, and level of protection (Vesk and Westoby, 2004; Deng et al., 2013a; Qian et al., 2014; Chen et al., 2015a). Defoliation may affect types of buds differently depending on their positioning and activation sensitivity (Huhat et al., 2000; N’Guessan and Hartnett, 2011). The release of apical dominance following decapitation may stimulate lateral bud outgrowth along the axis of a tiller (Cline, 1997). In *Schizachyrium scoparium*, an increase in defoliation frequency is associated with a significant increase in the proportion of extravaginal buds and a decrease in the proportion of intravaginal buds, resulting in a more spread-out, prostrate growth form (N’Guessan and Hartnett, 2011). Therefore, changes in the bud bank composition may contribute to changes in clonal growth strategies among perennial grasses in response to defoliation (Qian et al., 2014).

Previous studies on bud banks under defoliation have focused on the bud banks at the community or plant guild level in terrestrial grasslands (Dalgleish and Hartnett, 2009; Qian et al., 2014; VanderWeide and Hartnett, 2015). Clonal macrophytes, which are a common feature of wetland habitats, are grazed by herbivorous waterfowl and domestic livestock (Smith et al., 2012; Mesa et al., 2015). The way in which clonal macrophytes adjust the demographic features of bud banks, such as bud density and composition, to regulate population dynamics in response to defoliation has not been studied in wetlands.

In the present study, we investigated the effects of repeated defoliation on density, composition, and biomass of bud banks in the wetland sedge *Carex brevicuspis* C. B. Clarke, an important forage species for cattle and migratory birds, in the Dongting Lake wetlands, China. Belowground bud banks contribute almost 100% of the aboveground shoot recruitment in mature populations of *C. brevicuspis* (Deng et al., 2015). The plant produces two types of rhizome buds: short rhizome buds (SRB), which form clumping ramets, and long rhizome buds (LRB), which form spreading ramets (Chen et al., 2011, 2014; Deng et al., 2013b), resulting in a combined growth form. Our hypotheses were (1) that repeated defoliation would result in a decrease in the density and biomass of rhizome buds in *C. brevicuspis* and (2) that repeated defoliation in *C. brevicuspis* would produce a higher proportion of LRB and a lower proportion of SRB to promote a more spread-out growth form in order to avoid grazers. To test these hypotheses, we investigated the temporal dynamics of the shoot population and bud banks by sampling the aboveground shoot populations and the belowground bud banks for three defoliation frequencies (none, monthly, and bimonthly) over two consecutive years in the Dongting Lake wetlands.

## Materials and Methods

### Study Site

Dongting Lake (28°30′–30°20′N, 111°40′–113°10′E), the second largest freshwater lake in China, is located in the northern part of Hunan Province. It lies in a basin south of the Yangtze River and is connected to it by distributary channels. The surrounding wetlands are characterized by large seasonal fluctuations in the water level (up to 15 m) and are completely flooded June–October and exposed November–May. The mean annual temperature is 16.8°C, with hot summers (June–August, 27.3°C) and cold winters (December–February, 5.8°C) (Huang et al., 2013). The annual precipitation is 1382 mm, more than 60% of which falls between April and August. Our study site was located in the fence-enclosed monitoring plot (112°47′11.6″E, 29°29′14.3″N) of the Dongting Lake Station for Wetland Ecosystem Research from the Chinese Academy of Sciences. The number of days submerged in 2012 and 2013 were 171 and 167 days, respectively, and mean flooding depths were 2.60 ± 1.25 and 2.91 ± 1.15 m (mean ± SE) respectively, at our study site.

### Study species

*Carex brevicuspis* (Cyperaceae) is a perennial rhizomatous sedge found in eastern mainland China and Taiwan (Dai et al., 2010). The pseudoculm of the plant, consisting of a series of overlapping leaf sheaths, is usually 20–55 cm high. In the Dongting Lake wetlands, this species forms mono-dominant communities or is co-dominant with other *Carex* species. During the flood season (June–October), the *Carex* vegetation is completely submerged and the aboveground shoots senesce. *C. brevicuspis* shoots emerge immediately after flooding (November), growing to a standing crop before January (Chen et al., 2014). In January, the plants are relatively dormant and the shoots partially wither because of the low temperatures. New ramets sprout in March, after which the plants grow rapidly, flowering and fruiting from March to May, but producing only a few seedlings in the field (Chen et al., 2015c; Deng et al., 2015). *C. brevicuspis* populations outside natural reserves are grazed frequently by cattle, whereas those within natural reserves are grazed less frequently in the Dongting Lake wetlands.

### Experimental Design

Five sections of the lake shoreline dominated by *C. brevicuspis* were selected as study sites. The distance between each section was at least 200 m. In each section, three permanent quadrats (each 5 m × 5 m) were established parallel to the lake shoreline. The corners of each quadrat were marked by hammering durable plastic tubes into the soil. The distance between each quadrat was 5 m. One of three treatments (monthly defoliation, bimonthly defoliation, and no defoliation) was randomly assigned to each quadrat, with five replications of each treatment.

### Above- and Belowground Sampling

The experiment started on November 13, 2012 (after flooding). On that day, all ramets in the monthly and bimonthly quadrats were clipped to a height of 5 cm, to simulate cattle grazing. Thereafter, the plants in each quadrat were clipped according to the designed frequency (monthly, bimonthly, or not at all) during the non-flooding season.

Above- and belowground sampling occurred 2 months after the last bimonthly clipping and before the next clipping during the non–flooding season, January 2013–March 2014: in mid-November (1 week after flooding), mid-January (the coldest month), and mid-March (after spring sprouting). During each sampling, one square (50 cm × 50 cm) was randomly selected in each quadrat, for a total of 15 squares per sampling. In each square, all living (>50% green, potentially photosynthetically active) aboveground shoots were counted, clipped, and placed in plastic bags. Undisturbed soil within the squares was excavated to a depth of 15 cm using a shovel and stored in plastic bags (Chen et al., 2014, 2015b).

### Sample Processing

Belowground tissue samples were carefully washed to remove the soil while protecting the integrity of the rhizome buds. For each sampled square, the roots, LRB, SRB, and spacers (connections between ramets) were separated. The LRB were defined as the rhizome buds that grew horizontally further than 1 cm from the parent shoot (Bernard, 1990; Chen et al., 2011), whereas the SRB grew vertically and clumped around the parent shoot (Chen et al., 2014). As axillary buds in the rhizome nodes were inconspicuous (usually less than 1 mm in length), especially in short rhizomes, and contribute little to shoot populations in *Carex* species (Bernard, 1990; Deng et al., 2013b), only apical rhizome buds, which have the potential to sprout into ramets, were classified and counted (Chen et al., 2015c). The total rhizome bud (TRB) density was calculated as the sum of the SRB and LRB per m^2^. Aboveground shoots, roots, spacers, LRB, and SRB were dried separately in an oven at 80°C for 48 h before the dry weight was measured. The LRB or SRB biomass included, in each case, the apical bud and the attached rhizome. The total plant biomass was defined as the total dry weight of the shoots, roots, spacers, LRB, and SRB per m^2^. Total biomass per ramet was calculated as the total plant biomass divided by ramet density in each square. Biomass per TRB was calculated as the TRB mass divided by TRB density in each square.

### Data Analysis

The significance of differences in the height, density, and biomass of ramets, density, and biomass of rhizome buds, and proportion of SRB to TRB between defoliation treatments and sampling periods were evaluated by repeated analysis of variance (ANOVA), using defoliation frequency as a main factor and the sampling period as a repeated measure. Because some squares did not produce rhizome buds in March (i.e., the TRB density was zero), we did not analyze the differences in the proportion of SRB to TRB density between defoliation treatments for that month. Multiple comparisons of the means of plant traits under three defoliation frequencies at each sampling period were performed using Tukey’s honest significant difference (HSD) test at a 0.05 significance level. If necessary, the data were square root- or log_10_-transformed to reduce the variance heterogeneity, and the homogeneity was tested using Levene’s test. The data were expressed as the mean ± standard error (SE) and *p* < 0.05 was considered significant. All statistical analyses were performed using the statistical software SPSS V15.0 (SPSS Inc., USA).

## Results

### Ramet Height and Density

**FIGURE 1:**
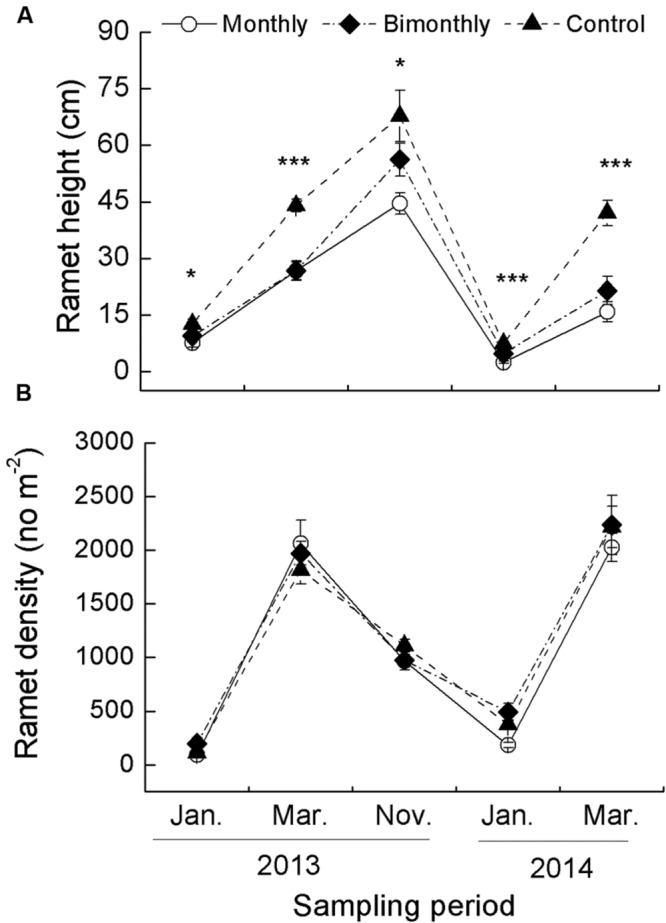
**Ramet height **(A)** and density **(B)** of *Carex brevicuspis* populations for three defoliation frequencies, January 2013–March 2014.** The data are expressed as the mean ± standard error (SE). ^∗^*p* < 0.05, ^∗∗∗^*p* < 0.001.

The ramet height was significantly affected by the defoliation frequency and sampling time, with significant interactions between both factors (**Table 1**). The monthly and bimonthly defoliation treatments significantly decreased the ramet height for all samplings January 2013–March 2014, with the exception of a non-significant decrease in January and November 2013 for the bimonthly defoliation treatment (**Figure 1A**). The ramet density was significantly affected by the sampling time but not by the defoliation frequency (**Table 1**; **Figure 1B**).

**Table 1:** Summary of repeated ANOVAs on plant traits in *Carex brevicuspis* populations for three defoliation frequencies January 2013–March 2014 (*F* and *P*-values).

Variable	Defoliation frequency (D)	Sampling time (S)	D × S
Ramet height	19.69ˆ***	188.35ˆ***	4.68ˆ**
Ramet density	1.23ˆns	149.53ˆ***	0.72ˆns
Shoot mass per ramet	48.20ˆ***	18.77ˆ***	11.04ˆ**
Root mass per ramet	7.05ˆ**	112.50ˆ***	3.41ˆns
Total biomass per ramet	7.45ˆ**	111.26ˆ***	3.48ˆns
SRB density	0.57ˆns	89.05ˆ***	3.10ˆ*
LRB density	0.57ˆns	17.40ˆ***	0.70ˆns
TRB density	0.57ˆns	90.03ˆ***	2.3ˆns
SRB proportion^a^	1.17ˆns	9.38ˆ**	2.32ˆns
Biomass per SRB	0.98ˆns	1.62ˆns	1.14ˆns
Biomass per LRB	3.06ˆns	30.76ˆ**	4.16ˆ**
Biomass per TRB	0.33ˆns	16.18ˆ***	3.14ˆ*

d.f.	2	4	8

*SRB, LRB, and TRB correspond to short, long, and total rhizome buds, respectively.*

*^∗∗∗^*p*< 0.001, ^∗∗^*p* < 0.01, ^∗^*p* < 0.05, ^ns^*p* > 0.05.*

*^a^d.f. for sampling time (S) is 2 and D × S is 4.*

### Shoot, Root, and Total Biomass Per Ramet

**FIGURE 2:**
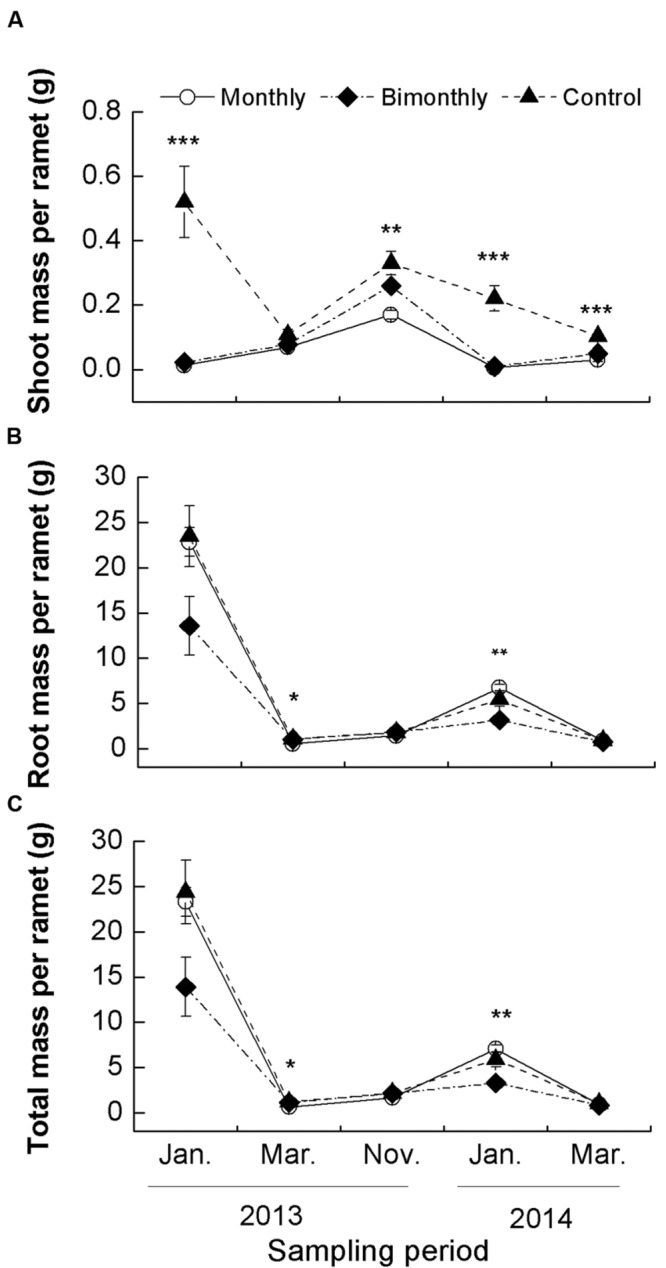
**Shoot **(A)**, root **(B)**, and total **(C)** mass per ramet of *C. brevicuspis* populations for three defoliation frequencies, January 2013–March 2014.** The data are expressed as the mean ± standard error (SE). ^∗^*p* < 0.05, *^∗∗^p* < 0.01, ^∗∗∗^*p* < 0.001.

Shoot mass per ramet was significantly affected by defoliation frequency and sampling time, with significant interactions between both factors (**Table 1**). Monthly defoliation significantly decreased the shoot mass per ramet for all sampling times, except for a non-significant reduction in March 2013 (**Figure 2A**). Bimonthly defoliation significantly decreased the shoot mass per ramet for all sampling times, except for non-significant reductions in March and November 2013 (**Figure 2A**).

The root and total biomass per ramet were also significantly affected by the defoliation frequency and sampling time (**Table 1**). In March 2013, monthly defoliation decreased the root and total biomass per ramet, while bimonthly defoliation did not reveal a significant reduction (**Figures 2B,C**). In January 2014, bimonthly defoliation significantly reduced the root and total biomass per ramet (**Figures 2B,C**).

### SRB, LRB, and TRB Density

**FIGURE 3:**
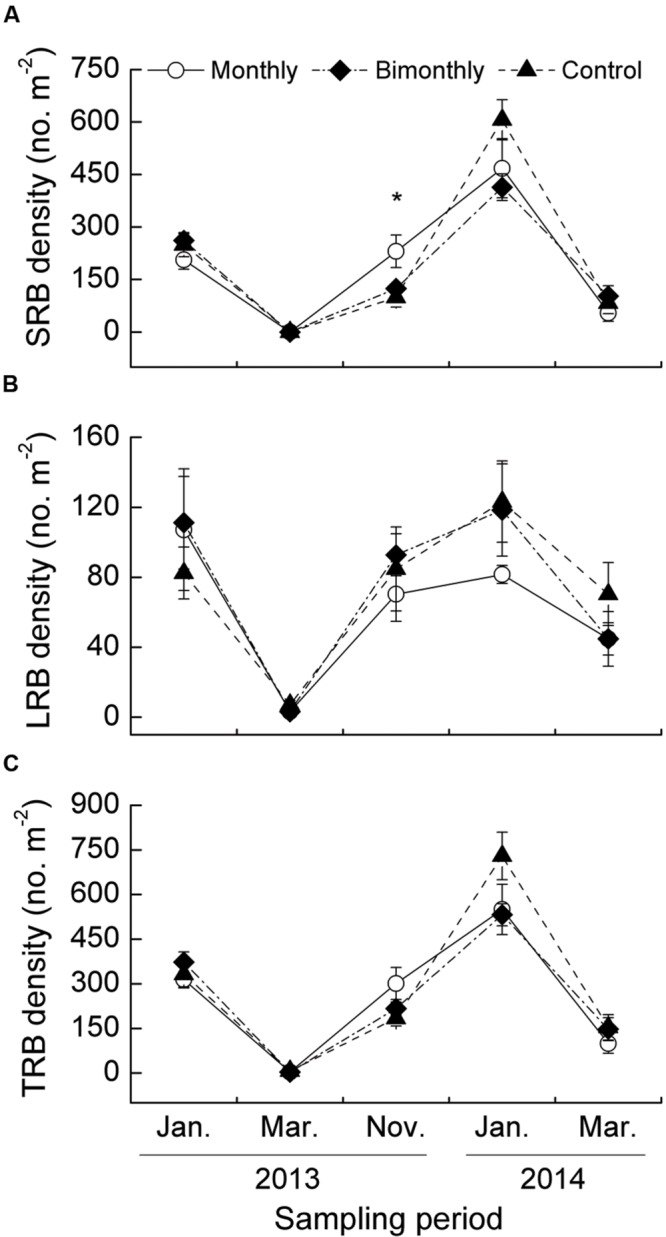
**Short rhizome bud (SRB, **A)**, long rhizome bud (LRB, **B)**, and total rhizome bud (TRB, **C)** density of *C. brevicuspis* populations for three defoliation frequencies, January 2013–March 2014.** Different scales are used on the *y*-axis. The data are expressed as the mean ± standard error (SE). ^∗^*p* < 0.05.

The SRB density was significantly affected by the sampling time, with significant interactions between the sampling time and defoliation frequency (**Table 1**). The monthly defoliation increased the SRB density in November 2013 (**Figure 3A**). The LRB and TRB density were significantly affected by the sampling time but not by the defoliation frequency (**Figures 3B,C**). The seasonal changes in the SRB, LRB, and TRB densities displayed similar trends for all treatments, peaking in January and dramatically decreasing in March (**Figures 3A–C**).

**FIGURE 4:**
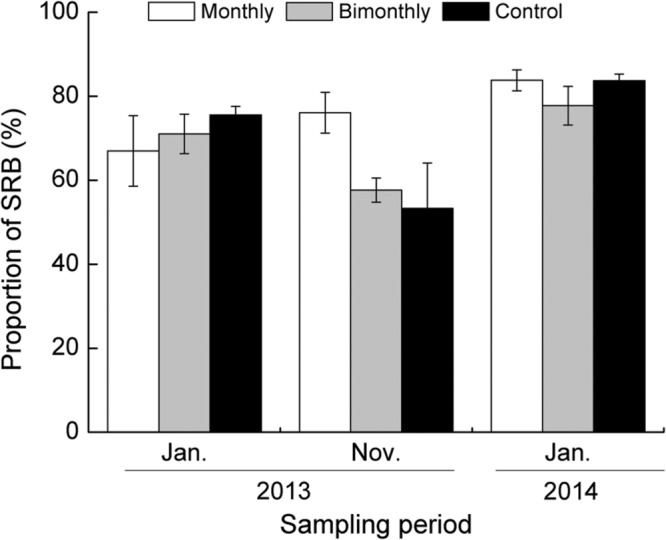
**Proportion of short rhizome buds (SRB) to total rhizome buds (TRB) of *C. brevicuspis* populations for three defoliation frequencies in January and November 2013 and January 2014.** The data are expressed as the mean ± standard error (SE). ^∗^*p* < 0.05.

The majority of buds among all treatments throughout the growing season were SRB (53.3–83.8%, **Figure 4**). The proportion of SRB to TRB was significantly affected by the sampling time but not by the defoliation frequency (**Table 1**; **Figure 4**).

### Biomass Per SRB, LRB, and TRB

**FIGURE 5:**
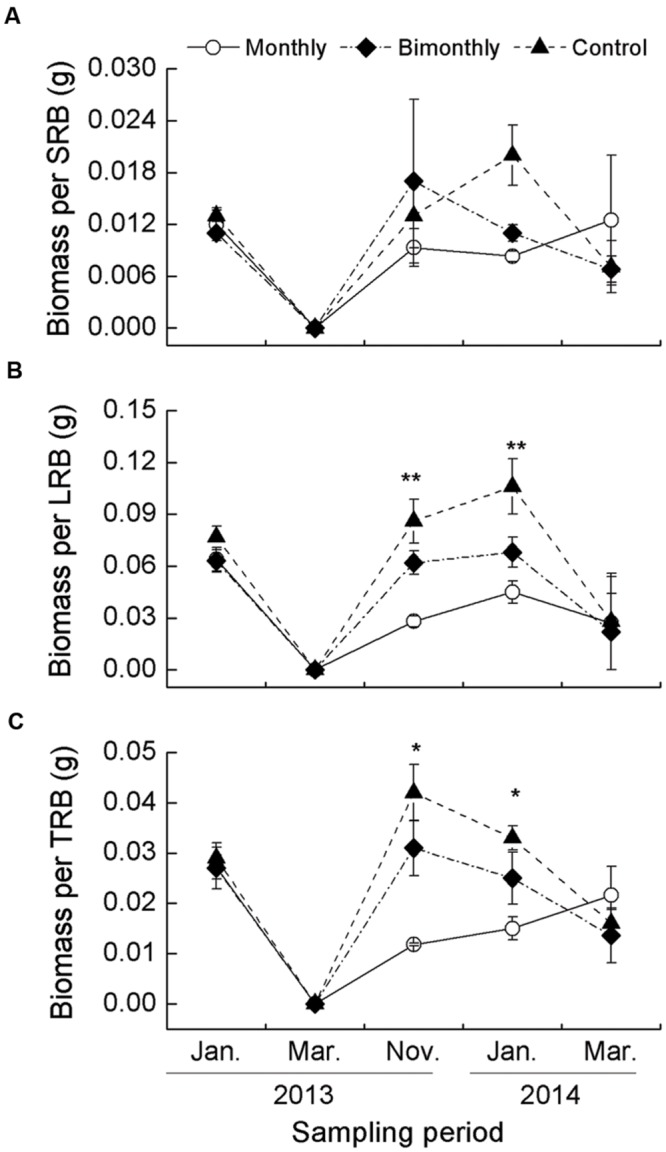
**Biomass per short rhizome bud (SRB, **A)**, long rhizome bud (LRB, **B)**, and total rhizome bud (TRB, **C)***C. brevicuspis* populations for three defoliation frequencies, January 2013–March 2014.** The data are expressed as the mean ± standard error (SE). ^∗^*p* < 0.05, ^∗∗^*p* < 0.01

The biomass per SRB was not significantly affected by defoliation frequency and sampling time (**Table 1, Figure 5A**). The biomass per LRB and TRB were significantly affected by the sampling time, with significant interactions between the sampling time and defoliation frequency (**Table 1**). Monthly defoliation decreased the biomass per LRB and TRB in November 2013 and January 2014, but bimonthly defoliation was not associated with a significant reduction (**Figures 5B,C**).

## Discussion

Repeated defoliation did not change the seasonal dynamics of the bud bank density of *C. brevicuspis*, which peaked in January and was lowest in March for all treatments. In addition, the TRB density of *C. brevicuspis* did not decrease significantly under repeated defoliation for 2 years (a total of 10 times for the monthly defoliation treatment). Therefore, our first hypothesis—that repeated defoliation would result in a decrease of the bud bank density—was proven false.

As a large number of buds sprout to replace the shoot population after defoliation, the number of dormant buds in the bud bank should decline after defoliation (Chen et al., 2015b). For example, grass bud banks decrease when the grass stem densities increase after grazing (Dalgleish and Hartnett, 2009). However, in the case of *C. brevicuspis*, which is a non-stem species, apical meristems were able to survive defoliation, as they are close to the ground (Chen et al., 2014). Defoliated ramets usually add buds as they regrow new leaves (Williams and Briske, 1991; Ott and Hartnett, 2011). Therefore, there were no significant differences in ramet or TRB density between defoliation treatments.

Nevertheless, the ramet height and the shoot, root, and rhizome bud biomass of *C. brevicuspis* decreased significantly under repeated defoliation. The results indicated that *C. brevicuspis* produces small-sized ramets and rhizome buds in response to repeated defoliation. Intense defoliation, which removes most of the photosynthetic tissues, usually reduces the plant’s growth and results in a smaller amount of carbon being stored (Ferraro and Oesterheld, 2002; Li et al., 2004; Zhao et al., 2008; Esmaeili et al., 2009; Liu and Li, 2010). The capacity of the plants to resprout from buds and grow after disturbances might be closely related to the carbon reserves in the perennial organs (Deng et al., 2013a), with bud production incurring significant opportunity and carbon allocation costs (Vesk and Westoby, 2004). Therefore, due to limited carbon storage after defoliation, *C. brevicuspis* might produce smaller individual ramet and rhizome buds.

Although the monthly defoliation treatment increased the density of short rhizomes in November 2013, the proportion of SRB to TRB did not change significantly during the study period. Therefore, our second hypothesis—that defoliation would promote a higher proportion of LRB and a lower proportion of SRB, creating a more spread-out growth form—was invalidated.

In *S. scoparium*, repeated defoliation was associated with a shift from vertical to more prostrate growth through changes in bud position, resulting in a greater proportion of tissue being inaccessible to herbivores (N’Guessan and Hartnett, 2011). In response to sedimentation or competitive stress, *C. brevicuspis* demonstrated a change from phalanx to guerrilla growth by producing a higher proportion of LRB and a lower proportion of SRB (Chen et al., 2011; Li et al., 2015). Previous studies indicated that long rhizomes enable tillers to escape from stressful microsites in a spatially heterogeneous habitat (de Kroon and Hutchings, 1995; Cheplick, 1997). However, the risk of being grazed may be equal for phalanx and guerrilla tillers, meaning that the grazing pressure may be homogenous for the *C. brevicuspis* tiller population. Furthermore, more energy may be required for the production of long rhizomes than for the production of short rhizomes (Cheplick, 1997). After defoliation, plants that allocate energy to produce long connections may be less competitive than plants that produce a dense population of ramets with short connections (Benot et al., 2009).

The maintenance of a large bud bank after repeated defoliation may contribute to the regeneration and persistence of *C. brevicuspis* populations in disturbed habitats. In *C. brevicuspis*, a large bud bank may confer greater ability to recover from severe damage than a small bud bank (Tuomi et al., 1994; Klimešová and Klimeš, 2007), potentially increasing the rates of shoot population recovery after disturbance (Dalgleish and Hartnett, 2009; Chen et al., 2015b). In addition, a large bud bank may increase the ability of clonal plants to respond to resource pulses such as increased precipitation or nutrient concentrations after disturbances (Dalgleish and Hartnett, 2006).

However, persistent grazing for many years may gradually deplete the amount of carbon stored and the bud banks, reducing the species’ capacity for recovery (N’Guessan and Hartnett, 2011; Qian et al., 2014). The present study also indicated that monthly defoliation reduced the biomass of rhizome buds and total biomass per ramet of *C. brevicuspis*, potentially affecting bud density in the long-term. Furthermore, small ramets and rhizomes of a *C. brevicuspis* population may be susceptible to invasion and replacement by exotic species (Dalgleish and Hartnett, 2009). The SRB and TRB density of *C. brevicuspis* were higher in January 2014 than in January 2013, especially in the control treatment, indicating inter-annual variation in bud bank density. Bud bank demography may be influenced by environmental factors such as soil water status and precipitation (Dalgleish and Hartnett, 2006; Deng et al., 2013b). The *C. brevicuspis* population that we studied was located in a natural reserve that experienced less herbivory than populations outside natural reserves. Tolerance to defoliation could differ among populations that have different histories of exposure to herbivores (Lu and Ding, 2012). Further investigation should include populations outside natural reserves and clarify the long-term effects of defoliation on bud banks of *C. brevicuspis*.

## Conclusion

Our study demonstrated that the density, composition, and seasonal dynamics of bud banks did not change significantly in response to monthly or bimonthly defoliation for 2 years. Bud banks of *C. brevicuspis* appear to follow a conservative reproductive strategy and were tolerant to grazing. However, repeated defoliation significantly reduced the plant size and the amount of carbon stored in the rhizomes. Long-term, frequent defoliation could have a negative effect on bud bank density.

## Author Contributions

X-SC and Y-HX wrote the manuscript and executed the technical assays and statistical analysis. X-SC and Y-HX designed the experiment and edited the manuscript text. Z-MD, FL, Z-YH, and CW contributed to data collection and interpretation. All authors reviewed the manuscript.

## Conflict of Interest Statement

The authors declare that the research was conducted in the absence of any commercial or financial relationships that could be construed as a potential conflict of interest.
